# Network Modeling for Post-Entry Management of Invasive Pest Species in the Philippines: The Case of the Colorado Potato Beetle, *Leptinotarsa decemlineata* (Say, 1824) (Coleoptera: Chrysomelidae)

**DOI:** 10.3390/insects14090731

**Published:** 2023-08-29

**Authors:** Billy Joel M. Almarinez, Divina M. Amalin, Kathleen B. Aviso, Heriberto Cabezas, Angelyn R. Lao, Raymond R. Tan

**Affiliations:** 1 Department of Biology, De La Salle University, Manila 0922, Philippines; 2 Department of Chemical Engineering, De La Salle University, Manila 0922, Philippines; kathleen.aviso@dlsu.edu.ph (K.B.A.); raymond.tan@dlsu.edu.ph (R.R.T.); 3 Research Institute of Applied Earth Sciences, University of Miskolc, 3515 Miskolc, Hungary; heriberto.cabezas@uni-miskolc.hu; 4 Department of Mathematics and Statistics, De La Salle University, Manila 0922, Philippines; angelyn.lao@dlsu.edu.ph

**Keywords:** ecological network analysis, food webs, ecological engineering, invasive species, Colorado potato beetle, potato

## Abstract

**Simple Summary:**

Crop switching is an important climate change adaptation strategy. New crops may need to be cultivated to provide food security as traditional staple crops become less suited to the changing climate in the bread baskets of many countries. For example, potato farming in the Philippines is being scaled up to supplement the cultivation of rice to meet the needs of a growing population. Since new crops introduced for this purpose can also be vulnerable to invasive pests, it is necessary to develop methods for planning pest management strategies that consider the complex interactions that can occur in farm ecosystems. In this paper, we develop a graph theoretic model for assessing pest management options for the prospective case of the entry of the Colorado potato beetle in potato farms in the Philippines. Two biological control agents and use of chemical pesticides are considered as alternative strategies. The model results indicate that the biological control strategies outperform the use of chemical pesticides. The concurrent use of both biological control agents could be less effective due to competition between the two species.

**Abstract:**

Crop shifting is considered as an important strategy to secure future food supply in the face of climate change. However, use of this adaptation strategy needs to consider the risk posed by changes in the geographic range of pests that feed on selected crops. Failure to account for this threat can lead to disastrous results. Models can be used to give insights on how best to manage these risks. In this paper, the socioecological process graph technique is used to develop a network model of interactions among crops, invasive pests, and biological control agents. The model is applied to a prospective analysis of the potential entry of the Colorado potato beetle into the Philippines just as efforts are being made to scale up potato cultivation as a food security measure. The modeling scenarios indicate the existence of alternative viable pest control strategies based on the use of biological control agents. Insights drawn from the model can be used as the basis to ecologically engineer agricultural systems that are resistant to pests.

## 1. Introduction

Climate change poses a significant threat to long-term food security [1]. Agriculture is predicated on the predictable recurrence of weather conditions that are favorable for the cultivation of crops that humans need. As climate change gradually shifts such established and familiar patterns, weather conditions may cease to become suitable for the continued cultivation of established crops. Thus, crop shifting in anticipation of future climate is widely regarded as an important adaptation strategy.

Global climate change is a well-documented and evolving challenge [1] that will likely affect all aspects of human existence, including the spread, establishment, and impact of agricultural insect pests to new geographic regions. However, climate change has three distinct aspects: long-term trends, short-term fluctuations, and tipping points. Both long-term trends [1] and short-term fluctuations [2] are manifested by observed changes over time in temperature, precipitation, etc. In addition, dynamic complex systems such as climate may have tipping points [3,4]. These are points in time when the properties of the system abruptly change over a relatively short period, making adaptation difficult if not impossible.

Climate change affects both natural and managed ecosystems. Increases in the concentration of atmospheric CO_2_ and in temperature and alterations in the pattern of precipitation have impacted both crops and insect pests in agroecosystems. Skendžić et al. [5] described how an increased carbon-to-nitrogen ratio in plants, a possible consequence of elevated CO_2_ concentration in the atmosphere, can lead to greater rates of consumption by herbivorous insects, increased developmental time, and a consequent decrease in pest abundance. Temperature increases, on the other hand, can affect insect pests through an increase in the number of generations, expansion of their distribution range, increased survival rates for overwintering species, and possible desynchronization between the pest species and their natural enemies or their host plants. Abnormally heavy precipitation may decrease overwintering of insect pests or their natural enemies in temperate regions, while droughts may cause greater susceptibility to insect pest attacks in plants [5].

The impacts from invasive alien species, including invasive pests, can also be compounded by climate change. Extreme climatic events resulting from climate change—such as typhoons or hurricanes, extreme droughts, and floods—can transport invasive pest species to new areas and decrease the resistance of habitats to invasions. This in turn can lead to increased geographic distribution of invasive pest species, with the result that pest outbreak status may impact the society and the environment. Biological invasions are a major threat to food security and subsistence, with developing countries being more susceptible. Due to high levels of subsistence and smallholder farming, these countries often lack the ability and funds to prevent and manage biological invasions. The annual economic costs and management of biological invasions are in the billions of US$. For instance, in the Philippines, the invasion of the coconut scale insect *Aspidiotus rigidus* Reyne 1947 in 2010–2016 cost more than 400 million Philippine pesos (approximately USD 8 million) for the implementation of treatment interventions [6]. The joint impact of biological invasions and climate change should direct policy responses towards these two issues that will help identify invasive species that could become a threat in the future.

The Colorado potato beetle (CPB), *Leptinotarsa decemlineata* (Say, 1824), is a major pest of the potato, *Solanum tuberosum* Linnaeus, 1753. A native to North America, this invasive insect has rapidly invaded some areas in Central America, as well as Europe and Asia. Maximum entropy models have predicted that under climate change scenarios, CPB and its first identified host, *S. angustifolium* (Houst. ex Mill., 1768)*,* can expand their distribution to include new areas of invasion in South Africa, South America, Australia, and Southeast Asia [7]. The Philippines, an agricultural country in Southeast Asia where the potato is cultivated as a food crop, currently has 43 reported plant species belonging to the family Solanaceae [8]. Of these solanaceous species in the country, six have been reported as hosts to *L. decemlineata*, including: the chili peppers, *Capsicum frutescens* Linnaeus, 1753 and *C. annuum* Linnaeus, 1753; the tomato, *S. lycopersicum* Linnaeus, 1753; the eggplant, *S. melongena* Linnaeus, 1753; the potato, *S. tuberosum*; and tobacco, *Nicotiana tabacum* Linnaeus, 1753 [9]. The latter two species are grown as cash crops in some northern provinces in the Philippines [10,11]. The potato, which has potential as a food security crop for climate change resilience in the Philippines [12], is therefore under major threat should climate change increase the chances of invasion by the destructive CPB.

Any deliberate attempt at crop shifting to secure future food supply should also consider the threat posed by concurrent changes in the geographic range of pests. Modeling tools are useful for the analysis of complex interactions that may exist in ecosystems. The socioecological process graph (SEP-graph) is suitable for this purpose. It is based on the P-graph framework, a technique originally developed for the computer-aided design of industrial processes [13,14]. Recently, it has been adapted for modeling socioecological networks [15,16,17]. The SEP-graph methodology makes use of nodes representing biological species that transform mass and energy flows and produce agricultural goods and services. The graph-theoretic optimization feature of the SEP-graph can determine robust co-culture strategies by controlling the presence of key species. It was used as a tool to address food security issues by proposing levels of productivity that can be achieved with different ecological network structures [16]. In Almarinez et al. [15], the SEP-graph was used to demonstrate the optimal integration of pest management strategies of *A. rigidus* during outbreak episodes and their eventual management in the Philippines. This modeling approach shows huge potential in exploring optimal interactions that provide positive influences on population and community dynamics, especially in agricultural systems that may be subject to disturbances caused by pests.

P-graph is a technique that was originally developed for the computer-aided design of process networks in industrial plants. This problem is known as process network synthesis (PNS) [13]. It classifies problem elements into O-type nodes (operating unit/processes) and M-type nodes (materials) in a bipartite graph framework. The M-type nodes are further classified into raw materials (inputs), intermediates, and products (outputs). Arcs are used to signify relationships between the nodes representing processes and their material inputs and outputs. Since in a bipartite graph no two nodes of the same type can be connected by arcs, a relationship between any two processes is always mediated by at least one material. Similarly, any two materials can be linked to each other only via a common process. The five axioms of PNS (shown in the left column of Table 1) are the basis for the rigorous development of the P-graph methodology [14].

The P-graph framework is at its core a constrained combinatorial search algorithm for feasible networks [13]. These are networks which can produce defined outputs given the inputs and the available operating units in the networks. The algorithms underlying the P-graph framework are very general and applicable to a broader class of networks, provided the constraints can be expressed in terms appropriate for the study of the network of interest, ecosystems in our case. Table 1 lists analogous features of the P-graph for process engineering and of the SEP-graph for ecological analysis [17].

Despite the criticality of crop shifting as a strategy to improve food security in the face of climate change, there is limited literature on the use of network models to analyze the complex interactions that can occur among introduced crops, their pests, and the biological control agents that can be used to manage the latter. To address this research gap, this study develops a SEP-graph model for this purpose and applies it to the case of managing CPB infestation risk in new potato farms in the Philippines.

## 2. Materials and Methods

### 2.1. Model Development

**Figure 1:**
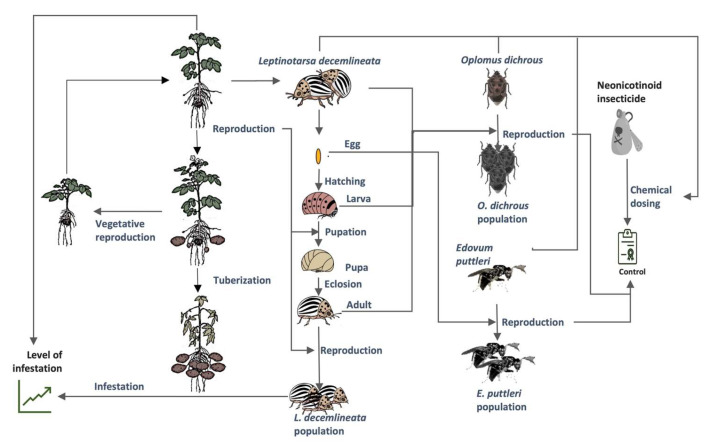
The potato–CPB agroecosystem. The line drawings of the potato plant growth stages were modified from Thornton (2020) [18].

**Figure 2:**
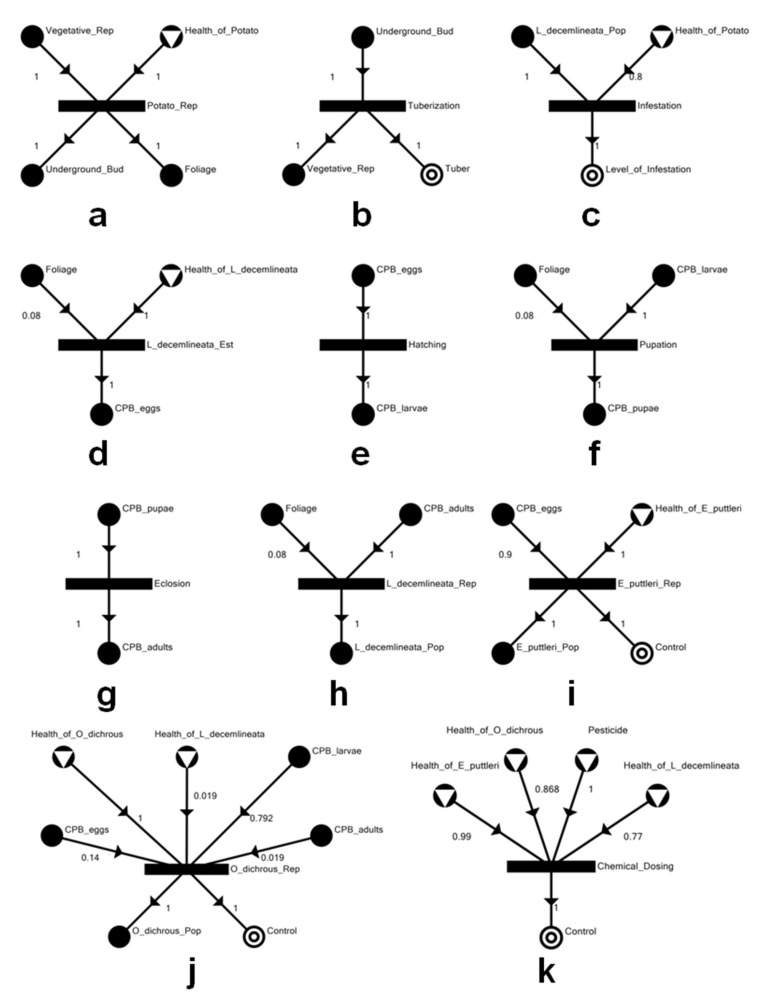
Individual processes that make up the potato agroecosystem: (**a**) potato reproduction; (**b**) tuberization; (**c**) *Leptinotarsa decemlineata* infestation; (**d**) establishment of invading *L. decemlineata*; (**e**) hatching of *L. decemlineata* eggs; (**f**) pupation of *L. decemlineata* larvae; (**g**) eclosion of *L. decemlineata* pupae; (**h**) reproduction of *L. decemlineata*; (**i**) reproduction of *Edovum puttleri*; (**j**) reproduction of *Oplomus dichrous*; and (**k**) dosing of pesticide. A circular vertex, or an M-type node, represents the stream of a raw material or an input to the network (with inverted triangle), an intermediate product or service (solid black), or a final product (double circle). The values indicate the rates of flow of the streams into and out of a process.

The implementation of the SEP-graph framework through P-graph Studio uses a graphical interface linked to a programming language to build models. For the case of the potato agroecosystem as illustrated in Figure 1, materials, species, and the state of their condition are represented by M-type nodes, and processes, relationships, or interactions between materials and species are represented by O-type nodes. The individual processes that occur within the ecosystem (solid rectangles) and the elements (circles) needed and generated from these processes are shown in Figure 2; these constitute the ecosystem of interest. Note that inputs coming from outside the ecosystem are shown as circles with an embedded triangle, inputs coming from inside the ecosystem are depicted as solid circles, and outputs going outside the ecosystem are represented by two concentric circles. The final outputs from the ecosystem include the (a) tuber, which represents the production of potatoes, (b) level of infestation which is an indicator of the proliferation of the CPB, and (c) control, which refers to pest control and can be implemented either by chemical (e.g., pesticides) or biological (e.g., natural enemies of CPB) means.

The processes depicted in SEP-graph form in Figure 2a–k represent the major biological functions needed for the agroecosystem to produce potatoes. Potato reproduction (Figure 2a) requires the health of the potato and vegetative reproduction to generate underground tubers and foliage. A similar interpretation can be made for the other processes. As in Almarinez et al. [15], the agroecological factors that were identified to be network components in the model were selected with the assumption of direct influence on potato tuber productivity in a scenario where *L. decemlineata* invades a potato-growing area in the Philippines as a consequence of climate change. Well-reported natural enemies of CPB, namely the egg parasitoid, *Edovum puttleri* Grissell, 1981 (Hymenoptera: Eulophidae) (Figure 2i), and the predatory stinkbug, *Oplomus dichrous* (Herrich-Schaeffer, 1838) (Hemiptera: Pentatomidae) (Figure 2j), were included in the model as biological control agents. These two natural enemies of the CPB have been reported to occur in tropical countries such as Mexico [9,19,20]. Thiamethoxam, a neonicotinoid that has been recommended for use against the CPB, was included to be the chemical pesticide (Figure 2k). A list of the P-graph labels and associated definitions of model materials are provided in Table 2.

The flow rates were derived from values reported for the average values of mortality across various *L. decemlineata* egg mass ages due to parasitism or probing by *E. puttleri* [21]; consumption rates of *O. dichrous* on CPB eggs, larvae, and adults [22]; mortality of *L. decemlineata* from toxicity rendered by thiamethoxam [23]; mortality of pentatomids exposed to thiamethoxam [24]; maximum potato yield loss to CPB without the use of control measures in Russia [25]; and defoliation threshold that can be tolerated by the potato plant [26,27]. These percentage values were translated into fuzzy values, with quantity type set to “capacity” in P-graph Studio for uniformity. Further details about the flow rates are provided in the Supplementary Table S1.

### 2.2. Network Analysis

Component process units of the model were assembled by P-graph Studio into a maximal structure using the maximal structure generation (MSG) algorithm. This maximal structure serves as the PNS problem’s master network [28]. The solution structure generation (SSG) algorithm was used to identify alternative networks that are structurally feasible, each of which could serve as the initial basis for an ecosystem configuration [15]. The accelerated branch-and-bound (ABB) algorithm was used to generate an optimal network after additional process data, such as flow rates, were specified. ABB-generated alternative solution structures and the represented real-world scenarios were examined on the basis of the resulting rates for the target outputs, namely consumable tuber production (“Tuber”), CPB infestation level (“Level_of_Infestation”), and pest control (“Control”).

## 3. Results

### 3.1. The Potato–CPB Agroecosystem Model

**Figure 3:**
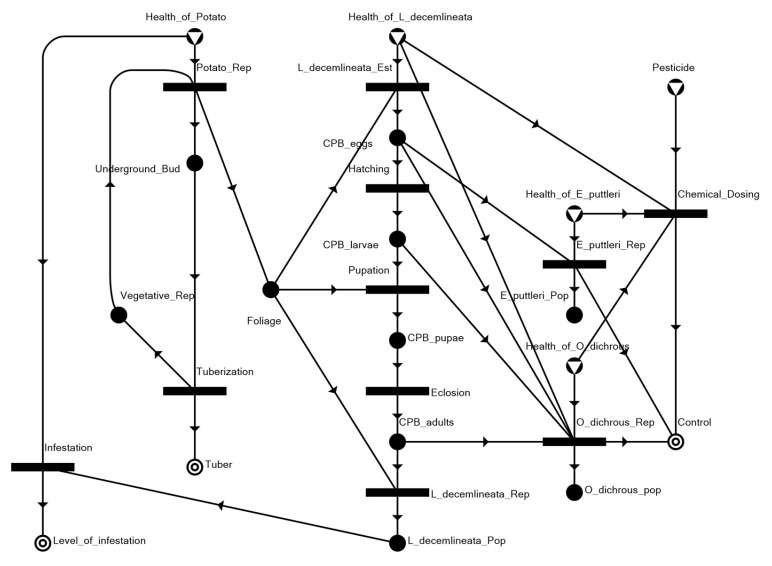
Maximal structure of the potato–CPB agroecosystem.

The overall model shown in Figure 3 is essentially constructed by appropriately joining the elements shown in Figure 2a–k into a network representing the entire agroecosystem.

With the assumption that only the selected agroecosystem factors would play a direct role in a pest invasion and management scenario, the following network linkages (i.e., trophic relationships) are highlighted in the network model: herbivory on the potato foliage by the invading *L. decemlineata* and by larvae for survival and sustenance towards pupation, and by adults whose reproduction establishes and sustains the infestation; predation by *O. dichrous* on CPB eggs, larvae, and adults; and parasitism by *E. puttleri* on CPB eggs.

### 3.2. Solution Structures and Represented Scenarios

**Figure 4:**
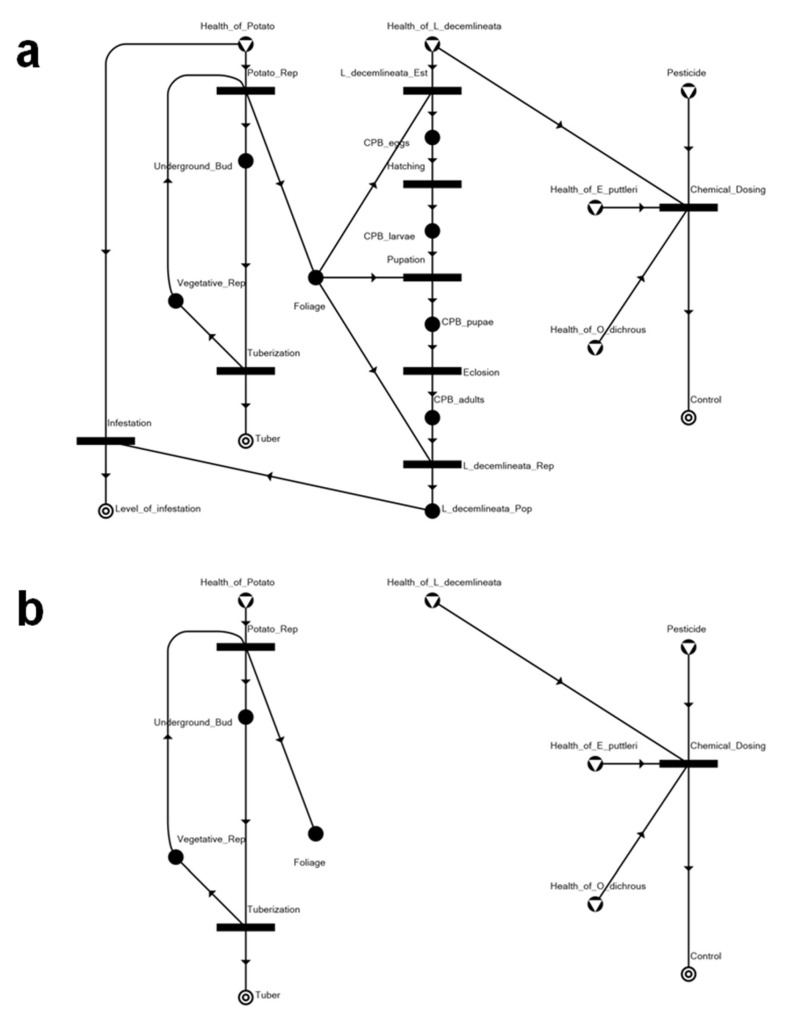
Network structures of potato–CPB pest management systems with application of chemical pesticide: (**a**) curative pesticide application (SS1) and (**b**) preventative pesticide application (SS4).

Applying the algorithms discussed above, we have generated 10 solution structures (SS), including a trivial null solution (SS10). These networks represent the alternative optimal equilibrium states of potato farm ecosystems under threat of *L. decemlineata* invasion and infestation. SS1 and SS4 present the curative and preventative control scenario of the ecosystem with the application of a chemical pesticide (Figure 4). With the preventative control shown in SS4, the tuber production is at its optimal state with no infestation. With the curative control shown in SS1, tuber production is kept at around 82% with a 23% level of CPB infestation (Table 3). In both solutions, both the predator and parasitoid released in the system as biocontrol agents totally succumb to the toxicity rendered by the pesticide.

**Figure 5:**
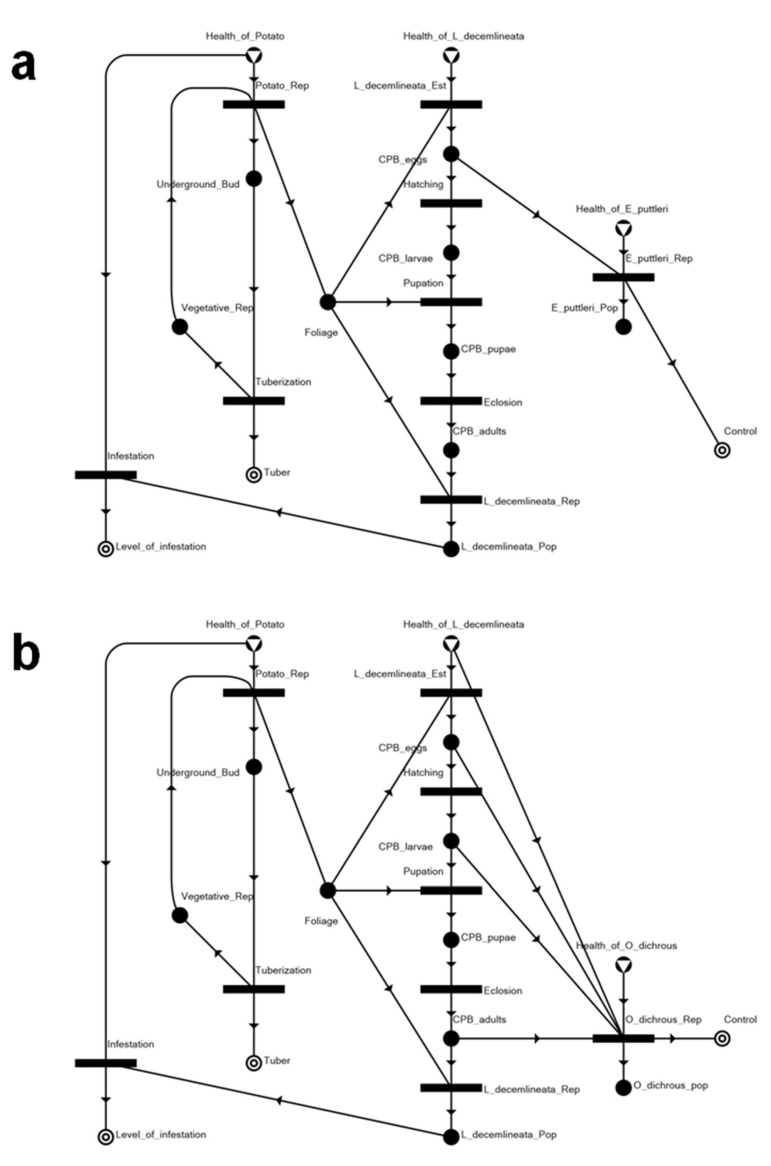
Network structures of potato–CPB pest management systems with biological control only: (**a**) *Edovum puttleri* as the biological control agent (SS2), and (**b**) *Oplomus dichrous* as the biological control agent (SS3).

SS2 and SS3 present biological control approaches to manage the infestation of CPB that has already established a population in the system (Figure 5). The difference between SS2 and SS3 is the biological control agent introduced into the potato agroecosystems. As shown in Table 3, the introduction of either biological control species will have the same effect/performance in terms of rendering overall pest control. However, using *O. dichrous* as a biological control agent results in slightly higher tuber production and a slightly lower CPB infestation level compared to having *E. puttleri* as the biological control agent.

**Figure 6:**
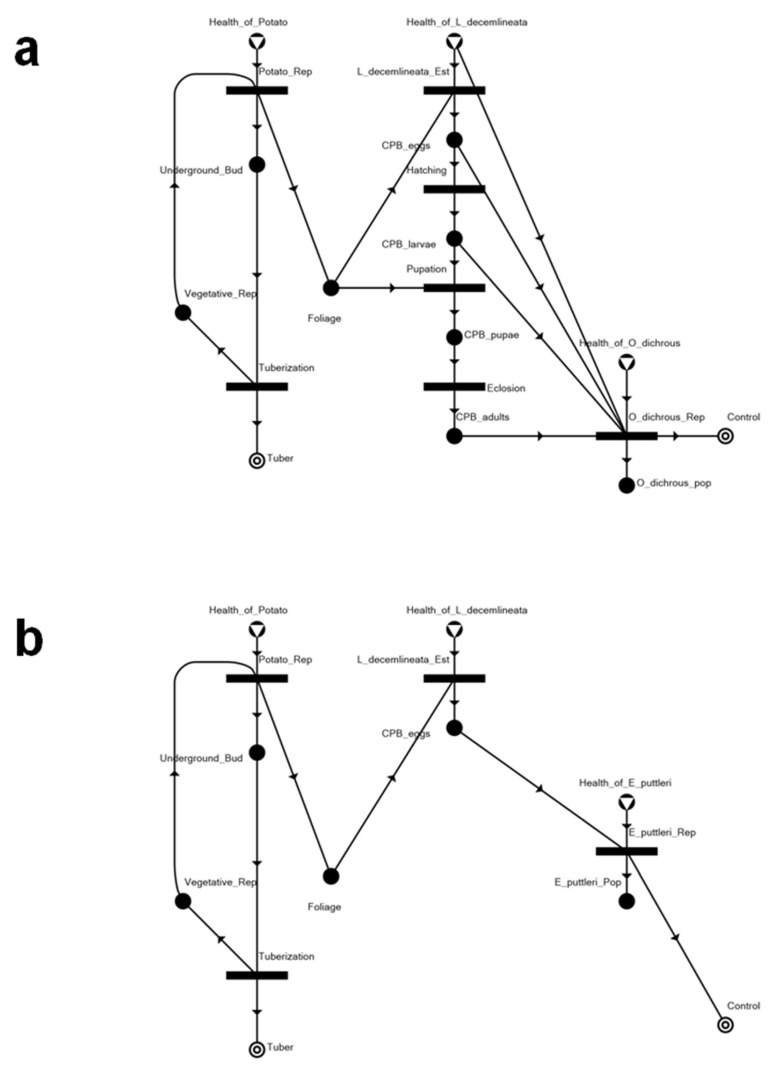
Network structures of potato–CPB pest management systems with preventative biological control approaches: (**a**) *Oplomus dichrous* as the biological control agent (SS5), and (**b**) *Edovum puttleri* as the biological control agent (SS6).

SS5 and SS6 also present preventative solutions that involve biological control (Figure 6). The biological control agents are introduced upon detection of the progeny of the invading CPB (all stages for *O. dichrous*, and eggs for *E. puttleri*). In both of these structures, it can be seen that there is no resulting infestation despite the introduction of the CPB into the system.

The healthy and uncontrolled potato agroecosystem is represented by SS7 and SS8, respectively. One of the solution structures, SS9, had all three insect species introduced into the system that do not have potato at all, only to succumb to toxicity rendered by the pesticide application.

## 4. Discussion and Conclusions

Starting with the P-graph framework, we developed a SEP-graph model of the potato agroecosystem under threat of invasion and infestation by the Colorado potato beetle. Optimization using the accelerated branch-and-bound (ABB) algorithm generated nine feasible solution structures, two of which represented chemically controlled infestation scenarios where the insecticide, thiamethoxam, is applied as either a curative (SS1) or a preventative (SS4) measure. The results from both measures may look good, achieving total (100%) overall control and between about 82 to 100 percent tuber production. However, intensive and sustained insecticide application has been well-reported as a major contributor to the development of insecticide resistance. Over the years, increasing resistance to neonicotinoids, including particularly thiamethoxam, has been observed in CPB populations from the United States [29,30,31] and from Canada [23,29]. Additionally, neonicotinoids render high toxicity on non-target species, particularly beneficials such as honeybees [32,33,34,35], and on predatory insects that are recognized as important biological control agents, such as the pentatomid *Podisus nigrispinus* (Dallas, 1851) [24]. Such non-target toxicity makes chemical control using thiamethoxam or other neonicotinoid commonly used for CPB control incompatible with the use of either the parasitic hymenopteran *Edovum puttleri* or the predatory pentatomid *Oplomus dichrous* as a biological control agent. SS1 and SS4 both show *E. puttleri* and *O. dichrous* totally succumbing to chemical dosing, further suggesting the aforementioned incompatibility between chemical and biological control approaches.

Interestingly, the ecosystems that only apply biological controls, represented in SS2 and SS3, generate higher yields of tuber production as compared to those that apply pesticides. This is what we considered the ideal scenario to control the attacks of the pests in a potato agroecosystem. These solution structures have the lowest level of infestation with the highest tuber production, with the difference between the two being the introduction of the biological control agent into the ecosystem. As an egg parasitoid, *E. puttleri* introduced in SS2 affects the proliferation of the CPB only at the egg stage. On the other hand, the *O. dichrous*, a predator, introduced in SS3, affects the proliferation of the CPB not only at the egg stage but also at the larval and adult stages, having more chances to attack the pests.

The other two solution structures representing biologically controlled networks, SS5 and SS6, suggest the introduction or release of either *O. dichrous* or *E. puttleri* as biological control agents for preventative management so that the invading CPB could be prevented from population establishment that would lead to a major infestation of potato plants in the agroecosystem. Either solution may be feasible if (a) either biological control agent can be easily imported from countries where they are mass-reared, and (b) proactive surveillance and monitoring systems are in place. Both *O. dichrous* and *E. puttleri* have been reported to thrive in tropical regions [19,20]; hence, their use for classical biological control of the CPB in case the pest invades the Philippines should be seriously considered and explored.

Note that no solution structure with both *E. puttleri* and *O. dichrous* in the agroecosystem resulting in overall control was generated and identified when the model was optimized using the ABB algorithm. This absence of such a solution may be the effect of possible mutual interference that could occur when both predator and parasitoid are present. Once either biological control agent is established, there are no incremental gains from introducing the other one. Egg parasitism by *E. puttleri* may result in a reduction in CPB eggs that can hatch into larvae, the most preferred prey stage for *O. dichrous* [22]. On the other hand, predation by *O. dichrous* on CPB eggs, larvae, and adults may lead to a reduction in the CPB eggs available for the reproduction of *E. puttleri*. The interaction between these two natural enemies of the CPB, when introduced in the same potato agro-ecosystem, is worth investigating via laboratory and controlled field studies.

Among the solution structures generated by the ABB algorithm was SS9, which represents a scenario where the CPB could be introduced without potato in the system. While this particular network appears trivial because of the absence of potato, introduction and establishment of an invasive insect species is always possible if it is polyphagous and if alternate hosts already occur in the area of invasion. Thus, the role of and possible effects on alternate plant hosts in the invasion and population establishment of the CPB in an agroecosystem should not be overlooked. The CPB has been reported to have at least eight alternate host plant species [9]. Aside from the potato, seven other plant species belonging to the family Solanaceae have been confirmed to be fed on by the CPB [36].

The Philippines is among the areas predicted to be suitable for the occurrence of *L. decemlineata* under present and future climate conditions, particularly the northwestern parts of Luzon Island which has been identified via Maxent modelling as high-risk for CPB invasion [7]. Therefore, potato-growing regions in the country, especially those in the Cordillera region in the northwestern part of Luzon, are at a considerable risk of CPB invasion under climate change. In addition to climate, the occurrence of alternate plant hosts may increase this invasion risk. The Philippines is home to at least forty-three (43) solanaceous species, six (6) of which are reported to be hosts of *L. decemlineata*, namely the tabasco pepper, the chili pepper, the tobacco plant, the tomato, the eggplant, and the potato [8]. All six plant species are considered in the country as valuable agricultural commodities, with tobacco actually being a cash crop in the northern provinces of Luzon Island [11]. In addition to the possible role that these solanaceous species may play in the invasion and establishment of *L. decemlineata* in the Philippines, the ecological and economic consequences of infestation by CPB on these alternate host plants should also be seriously investigated.

The entry of invasive plant pest species is very difficult to prevent due to several pathways or routes of introduction, which can be classified as natural or manmade. Natural pathways could be through wind, water currents, and other forms of natural dispersal that can bring species to a new habitat; manmade are those associated with human activity, either intentional or unintentional. Therefore, success of interception is quite low, increasing the chance of pest outbreak. To mitigate the outbreak from invasive pest species, post-entry management should be in place before the entry into the new area.

The network effects of introducing biological control agents may be difficult to predict due to complex interactions among the components of agricultural ecosystems. Some of these effects may be counterintuitive and hence lead to unintended consequences. Ecological network modelling techniques such as SEP-graph can be used to improve predictions about the efficacy of interventions. In this study, the result of the SEP-graph illustrates the scenario of preventing outbreak—how to manage and prevent the spread and establishment of CPB—once it enters the Philippines. The SEP-graph was used to generate alternative equilibrium states of potato farms under different infestation and control conditions. Biological control with either the predatory or parasitoid agent was found to be superior to chemical control in terms of maintaining crop yield. However, no evidence was found of any beneficial effect from using both biological control agents at the same time. Their simultaneous presence may result in mutual interference, as suggested by our model. While this potential mutual interference between the two natural enemies of CPB remains a hypothesis at this point, SEP-graph modelling has provided a theoretical basis for the use of just one natural enemy species if classical biological control were to be considered as a post-entry strategy against the CPB in the likelihood of climate-influenced invasion.

In practice, the calibration of ENA models is often hampered by limited data availability [37]. Alternative approaches have been proposed to allow models to be developed using qualitative information drawn from expert estimates. For example, techniques have been demonstrated on the basis of fuzzy cognitive maps (FCMs), a semiquantitative modelling tool first proposed by Kosko [38]. This approach was shown to be a workable alternative to purely quantitative ENA models for modelling ecosystems with limited data; applications include analysis of pest management strategies [39] and nutrient flows [40]. As a matter of best practice, building FCM models requires calibration with expert knowledge coupled with sensitivity analysis. A similar semiquantitative approach can be used in the SEP-graph framework [15]. This modelling technique can be readily applied to managing other crop pests, as it has the advantage of being usable under conditions of data scarcity. Subjective expert estimates can be used as proxies for hard data in such cases. However, the modelling technique itself does not consider ecosystem dynamics. Future variants or hybrid approaches can be used to model the behavior of agricultural systems over time.

## Figures and Tables

**Table 1:** The five original axioms of PNS (left column) [14] and the equivalent formulation for ecosystems (right column) [17].

Process Engineering	Socio-Ecological Systems
(S1) Every final product is represented in the structure	(SE1) There should be at least one well-defined terminal ecosystem service in the ecosystem structure.
(S2) A material represented in the structure is a raw material if and only if it is not an output of any operating unit represented in the structure	(SE2) An ecosystem service represented in the structure is exogenous if and only if it is not an output of a functional unit defined in the ecosystem structure.
(S3) Every operating unit represented in the structure is defined in the synthesis problem	(SE3) Every ecosystem functional unit in the ecosystem structure is well defined.
(S4) Any operating unit represented in the structure has at least one path leading to a product	(SE4) Any ecosystem functional unit has at least one path leading to a terminal ecosystem service.
(S5) If a material belongs to the structure, it must be an input to or output from at least one operating unit represented in the structure	(SE5) If an ecosystem service belongs to the ecosystem structure, it must be an input to or output from at least one ecosystem functional unit represented in the structure.
Assumption: capital is available to pay for operating units and operating costs.	Assumption: energy is available to keep the ecosystem structure functioning
Goals: (1) meet production goal at (2) minimum cost for the structure and operation	Goals: (1) meet ecosystem services goal; and (2) minimize a cost metric (e.g., money, ecological footprint, energy, etc.) for the structure, management, functioning, and operations of the ecosystem services

**Table 2:** P-graph labels and associated definitions of model materials.

Material/Unit	Description
Chemical_Dosing	Chemical control strategy against *Leptinotarsa decemlineata* (CPB)
Control	Overall pest control
CPB_adults	Adult CPB
CPB_eggs	Eggs of CPB
CPB_larvae	Larvae of CPB
CPB_pupae	Pupae of CPB
E_puttleri_Pop	*Edovum puttleri* population
E_puttleri_Rep	Reproductive capacity of *E. puttleri*
Eclosion	Eclosion of CPB pupae
Foliage	Foliage of the potato plant
Hatching	Hatching of CPB eggs
Health_of_E_puttleri	Overall health of *E. puttleri*
Health_of_L_decemlineata	Overall health of CPB
Health_of_O_dichrous	Overall health of *Oplomus dichrous*
Health_of_Potato	Overall health of the potato plant
Infestation	Infestation capacity of CPB on the potato plant
L_decemlineata_Est	Establishment of invading CPB
L_decemlineata_Pop	CPB population
L_decemlineata_Rep	Reproductive capacity of CPB on the host potato plant
Level_of_Infestation	Observable level of infestation by CPB on the host potato plant
O_dichrous_Pop	*O. dichrous* population
O_dichrous_Rep	Reproductive capacity of *O. dichrous*
Pesticide	Thiamethoxam
Potato_Rep	Reproduction of the potato plant
Pupation	Pupation of CPB larvae
Tuber	Yield of consumable potato tubers
Tuberization	Formation of tubers by the potato plant
Underground_Bud	Underground buds of the potato plant
Vegetative_Rep	Vegetative reproduction of the potato plant via tubers not for human consumption

**Table 3:** ABB-generated solution structures and the represented pest management scenarios.

Solution Structure	Represented Scenario	Tuber Production	Level of Infestation	Control
SS1	Chemically controlled system, with two biological control agents eradicated as well	0.816	0.23	1
SS2	Biologically controlled system with *Edovum puttleri*	0.920	0.1	1
SS3	Biologically controlled system with *Oplomus dichrous*	0.976	0.03	1
SS4	Insecticide applied prior to *Leptinotarsa decemlineata* (CPB) establishment, eradicating either biological control agent	1	N.A.	1
SS5	Biologically controlled system with release of *E. puttleri* upon detection of eggs from invading CPB	1	N.A.	1
SS6	Biologically controlled system with release of *O. dichrous* upon the occurrence of immature and mature progeny of invading CPB	1	N.A.	1
SS7	System with uncontrolled CPB infestation	0.231	0.961538	N.A.
SS8	Completely healthy system, without CPB	1	N.A.	N.A.
SS9	System without potato, but with invading CPB and occurring natural enemies killed by insecticide	N.A.	N.A.	1
SS10	Null	N.A.	N.A.	N.A.

## Data Availability

Data used in the study, including the P-graph studio file, may be requested from either of the corresponding authors (B.J.M.A. or D.M.A.).

## Supplementary Materials

The following supporting information can be downloaded at: https://www.mdpi.com/article/10.3390/insects14090731/s1, Table S1: Flow rates and bases/references.
