# Supplementary material for: Network Modeling for Post-Entry Management of Invasive Pest Species in the Philippines: The Case of the Colorado Potato Beetle, Leptinotarsa decemlineata (Say, 1824) (Coleoptera: Chrysomelidae)
Source: Insects. 2023 Aug 29;14(9):731. doi: 10.3390/insects14090731 (PMC10532276; doi:10.3390/insects14090731)
Supplement: Supplementary file 1 [file insects-14-00731-s001.zip › insects-2457964-supplementary.pdf]

SUPPLEMENTARY MATERIAL

Table S1. Flow rates and bases/references

Component	Interaction	Flow Rate	Explanation	Basis/Reference
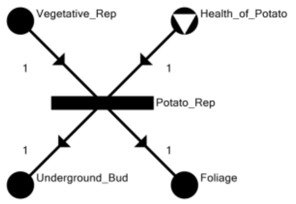 Potato Reproduction	Health_of_Potato → Potato_Rep	1.00	Reproductive capacity of the potato plant is expected to be most highly influenced by its overall health.	Assumed
	Vegetative_Rep → Potato_Rep	1.00	Maximal vegetative reproductive rate of the potato plant is expected to result to maximal reproduction of potato	Assumed
	Potato_Rep → Underground_Bud	1.00	Reproduction in potato involves production of underground buds.	Assumed
	Potato_Rep → Foliage	1.00	Reproduction in potato involves maintenance of foliage.	Assumed
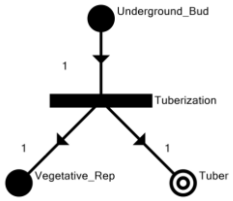 Tuberization	Underground_Bud → Tuberization	1.00	The underground bud undergoes tuberization to form tubers for vegetative reproduction.	Assumed
	Tuberization → Vegetative_Rep	1.00	Maximal tuberization rate results to maximal vegetative reproductive rate of the potato plant.	Assumed
	Tuberization → Tuber	1.00	Maximal tuberization rate results to maximal consumable potato tuber production.	Assumed
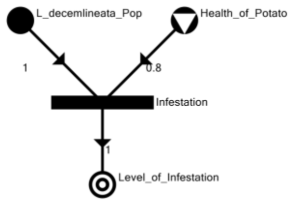 *Leptinotarsa decemlineata* Infestation	L_decemlineata_Pop → Infestation	1.00	Population establishment of *L. decemlineata* leads to infestation on the potato plant.	Assumed
	Health_of_Potato → Infestation	0.80	Maximum yield loss of 80% in potato can be caused by an infestation of *L. decemlineata* when control measures are not implemented.	Maharijaya & Vosman [25]
	Infestation → Level_of_infestation	1.00	The observable level of infestation of *L. decemlineata* on the potato plant is expected to be a function of the infestation capacity of the pest.	Assumed

<table>
<tr><td rowspan="2">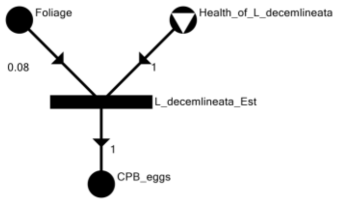

Establishment of Invading L. decemlineata</td><td>Foliage → L_decemlineata_Est</td><td>0.08</td><td>Establishment of invading L. decemlineata is estimated to require at least 8 percent of the foliage of the potato plant, or about one-third of 25% which is the reported defoliation threshold that can be tolerated by the potato plant.</td><td>Varenhorst et al. [26]; Ragsdale & Radcliffe [27]; assumed</td></tr>
<tr><td>L_decemlineata_Est → CPB_eggs</td><td>1.00</td><td>Oviposition would result from the establishment of the invading L. decemlineata.</td><td>Assumed</td></tr>
<tr><td rowspan="2">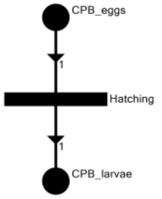

Hatching of CPB Eggs</td><td>CPB_eggs → Hatching</td><td>1.00</td><td>L. decemlineata eggs will undergo hatching.</td><td>Assumed</td></tr>
<tr><td>Hatching → CPB_larvae</td><td>1.00</td><td>Hatching of the CPB eggs will produce larvae.</td><td>Assumed</td></tr>
<tr><td rowspan="3">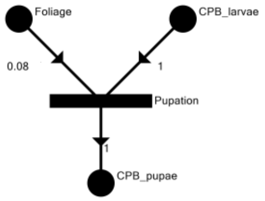

Pupation of CPB Larvae</td><td>Foliage → Pupation</td><td>0.08</td><td>Pupation of L. decemlineata larvae is estimated to require at least 8 percent of the foliage of the potato plant, or about one-third of 25% which is the reported defoliation threshold that can be tolerated by the potato plant.</td><td>Varenhorst et al. [26]; Ragsdale & Radcliffe [27]; assumed</td></tr>
<tr><td>CPB_larvae → Pupation</td><td>1.00</td><td>CPB larvae will undergo pupation.</td><td>Assumed</td></tr>
<tr><td>Pupation → CPB_pupae</td><td>1.00</td><td>Pupation of larvae will produce CPB pupae.</td><td>Assumed</td></tr>
</table>

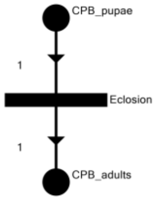<br><br>Eclosion of CPB Pupae	CPB_pupae → Pupation	1.00	CPB pupae will undergo eclosion.	Assumed
	Pupation → CPB_pupae	1.00	Eclosion of pupae will produce CPB adults.	Assumed
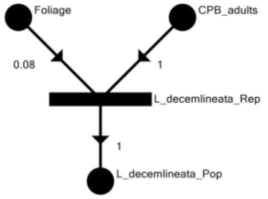<br><br>Reproduction of CPB	Foliage → L_decemlineata_Rep	0.08	Reproduction by *L. decemlineata* adults is estimated to require at least 8 percent of the foliage of the potato plant, or about one-third of 25% which is the reported defoliation threshold that can be tolerated by the potato plant.	Varenhorst *et al.* [26]; Ragsdale & Radcliffe [27]; assumed
	CPB_adults → L_decemlineata_Rep	1.00	CPB adults will mate and reproduce.	Assumed
	L_decemlineata_Rep → L_decemlineata_Pop	1.00	Maximal reproductive capacity of *L. decemlineata* leads to further population establishment of CPB on the host potato plant.	Assumed
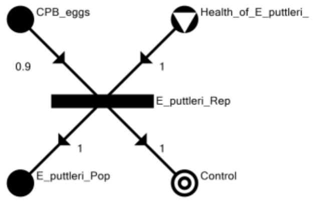<br><br>Reproduction of *Edovum puttleri*	CPB_eggs → E_puttleri_Rep	0.90	Average mortality of 90% in CPB egg masses resulting from parasitism or probing by *E. puttleri*	Lashomb *et al.* [21]
	Health_of_E_puttleri → E_puttleri_Rep	1.00	Maximum reproductive capacity of *E. puttleri* would require healthy parasitoids that can effectively search for and parasitize CPB eggs	Assumed
	E_puttleri_Rep → E_puttleri_Pop	1.00	Maximal reproductive capacity of *E. puttleri* leads to population establishment on the CPB-infested potato plant.	Assumed
	E_puttleri_Rep → Control	1.00	*E. puttleri* parasitism (reproduction) renders biological control of CPB.	Lashomb *et al.* [21]; assumed

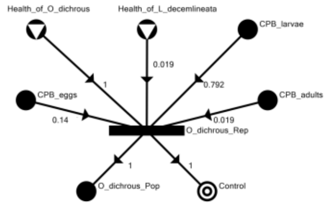 Reproduction of *Oplomus dichrous*	CPB_eggs → O_dichrous_Rep	0.14	95% overall CPB mortality x 14% CPB egg mortality resulting from the consumption by *O. dichrous* across developmental stages of the CPB	Drummond *et al.* [22]; assumed
	Health_of_O_dichrous → O_dichrous_Rep	1.00	Maximum reproductive capacity of *O. dichrous* would require healthy bugs that can effectively search for and prey on CPB eggs, larvae, and adults.	Assumed
	Health_of_L_decemlineata → O_dichrous_Rep	0.019	Consumption rate of *O. dichrous* on the invading adult CPB at 95% overall CPB mortality x 2% adult CPB mortality resulting from the consumption by *O. dichrous* across developmental stages of the CPB	Drummond *et al.* [22]; assumed
	CPB_larvae → O_dichrous_Rep	0.792	95% overall CPB mortality x 83.4% larval mortality CPB resulting from the consumption by *O. dichrous* across developmental stages of the CPB	Drummond *et al.* [22]; assumed
	CPB_adults → O_dichrous_Rep	0.019	95% overall CPB mortality x 2% adult CPB mortality resulting from the consumption by *O. dichrous* across developmental stages of the CPB	Drummond *et al.* [22]; assumed
	O_dichrous_Rep → O_dichrous_Pop	1.00	Maximal reproductive capacity of *O. dichrous* leads to population establishment on the CPB-infested potato plant.	Assumed
	O_dichrous_Rep → Control	1.00	*O. dichrous* predation (reproduction) renders biological control of CPB.	Drummond *et al.* [22]; assumed
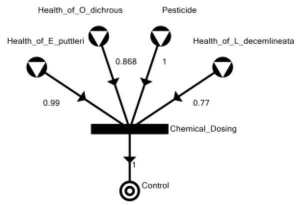 Chemical Pest Control	Pesticide → Chemical_Dosing	1.00	Chemical insecticides, particularly neonicotinoids such as thiamethoxam, have been used for control of CPB.	Scott *et al.* [23]
	Chemical_Dosing → Control	1.00	Neonicotinoid insecticide thiamethoxam renders population control of target and non-target insects.	Assumed
	Health_of_E_puttleri → Chemical_Dosing	0.99	*E. puttleri*, a parasitic hymenopteran, would be most likely as highly susceptible to neonicotinoid toxicity similar to how it was for other parasitic wasps such as *Comperiella calauanica*.	Almarinez *et al.* unpublished data on neonicotinoid toxicity in *C. calauanica*

	Health_of_O_dichrous → Chemical_Dosing	0.868	*O. dichrous* could exhibit susceptibility to thiamethoxam toxicity as *Podisus nigrispinus*, another predatory pentatomid, when exposed to treated plants or fed with treated prey.	Torres *et al.* [24]
	Health_of_L_decemlineata → Chemical Dosing	0.77	Mean thiamethoxam-induced mortality of CPB in 2011 in Canada	Scott *et al.* [23]
